# Amino-Acid Co-Variation in HIV-1 Gag Subtype C: HLA-Mediated Selection Pressure and Compensatory Dynamics

**DOI:** 10.1371/journal.pone.0012463

**Published:** 2010-09-01

**Authors:** Morgane Rolland, Jonathan M. Carlson, Siriphan Manocheewa, J. Victor Swain, Erinn Lanxon-Cookson, Wenjie Deng, Christine M. Rousseau, Dana N. Raugi, Gerald H. Learn, Brandon S. Maust, Hoosen Coovadia, Thumbi Ndung'u, Philip J. R. Goulder, Bruce D. Walker, Christian Brander, David E. Heckerman, James I. Mullins

**Affiliations:** 1 Department of Microbiology, University of Washington, Seattle, Washington, United States of America; 2 Department of Medicine, University of Washington, Seattle, Washington, United States of America; 3 Microsoft Research, Redmond, Washington, United States of America; 4 HIV Pathogenesis Program, Nelson Mandela School of Medicine, University of KwaZulu-Natal, Durban, South Africa; 5 Department of Pediatrics, Nuffield Department of Medicine, Oxford University, Oxford, United Kingdom; 6 Ragon Institute of MGH, MIT and Harvard, Massachusetts General Hospital and Harvard Medical School, Boston, Massachusetts, United States of America; 7 Howard Hughes Medical Institute, Chevy Chase, Maryland, United States of America; Institut Pasteur, France

## Abstract

**Background:**

Despite high potential for HIV-1 genetic variation, the emergence of some mutations is constrained by fitness costs, and may be associated with compensatory amino acid (AA) co-variation. To characterize the interplay between Cytotoxic T Lymphocyte (CTL)-mediated pressure and HIV-1 evolutionary pathways, we investigated AA co-variation in Gag sequences obtained from 449 South African individuals chronically infected with HIV-1 subtype C.

**Methodology/Principal Findings:**

Individuals with CTL responses biased toward Gag presented lower viral loads than individuals with under-represented Gag-specific CTL responses. Using methods that account for founder effects and HLA linkage disequilibrium, we identified 35 AA sites under Human Leukocyte Antigen (HLA)-restricted CTL selection pressure and 534 AA-to-AA interactions. Analysis of two-dimensional distances between co-varying residues revealed local stabilization mechanisms since 40% of associations involved neighboring residues. Key features of our co-variation analysis included sites with a high number of co-varying partners, such as HLA-associated sites, which had on average 55% more connections than other co-varying sites.

**Conclusions/Significance:**

Clusters of co-varying AA around HLA-associated sites (especially at typically conserved sites) suggested that cooperative interactions act to preserve the local structural stability and protein function when CTL escape mutations occur. These results expose HLA-imprinted HIV-1 polymorphisms and their interlinked mutational paths in Gag that are likely due to opposite selective pressures from host CTL-mediated responses and viral fitness constraints.

## Introduction

HIV is characterized by extensive genetic diversity, yet significant segments of the HIV-1 proteome are conserved across all subtypes [1], implying that there are limits to HIV variability: not all codons have been found to accept a mutation, and some replacements are only found in the presence of other mutations, exemplifying an evolutionary co-variation process among these amino acids (AA).

To assess co-variation robustly, Poon [2] and Carlson and their colleagues [3], [4], [5] developed phylogenetically-informed methods, which were designed to remove spurious associations stemming from shared ancestry and HLA linkage. A central tenet for assessing co-variation is the covarion principle, proposed by Fitch and Markowitz in 1970 [6], which states that ‘at any one point in time only a very restricted number of positions can fix mutations but that as mutations are fixed, the positions capable of accepting mutations also change so that examination of a wide-range of species reveals a wide range of altered positions. We define this restricted group as the concomitantly variable codons [and] suggest the term “covarions” to describe this particular set of codons.’

Experimentally, it has been shown that AA changes are often coupled: Yanofsky and colleagues first showed in 1964 that second-site compensatory mutations could suppress the deleterious effects of some mutations [7]. Since then, several genetic studies have revealed cooperative AA changes, including, for HIV patients on antiretroviral therapy, the development in Pol of drug resistance mutations (DRM) which were accompanied by the fixation of secondary mutations [8]–[15]. Examples of compensatory changes in HIV-1 Gag include the partial restoration of the fitness cost of the CTL escape mutation A163G (in the B*5703 KAFSPEVIPMF) by the S165N change within the epitope [16]; likewise, the fitness cost of the CTL escape mutation R264K (in the B*27 KRWIILGLNK) is partially restored by S173A [17], and the I147L in QAISPRTLNAW (QW11) mutation partially compensates the fitness cost associated with the escape mutation A146P [18].

To better understand the structural variability of HIV-1, and specifically the relationship between interlinked mutations and compensatory changes, we assessed patterns of co-variation in subtype C Gag in a cohort of chronically infected persons who had not received antiretroviral treatment at the time of analysis. Gag-specific CTL responses are considered important in the control of HIV replication [19], and certain Gag CTL escape mutations reduce viral fitness, thereby possibly promoting the containment of a partially-crippled virus [1], [16], [17], [20], [21]. Our study sought to build upon studies describing HLA-driven HIV-1 variation using phylogenetic correction [22], [23] by further analyzing co-variation pathways that have partially been described [4] in order to link these CTL escape mutations.

## Results

### Protective effect of CTL responses targeting conserved HIV-1 proteins, specifically Gag

**Figure 1:**
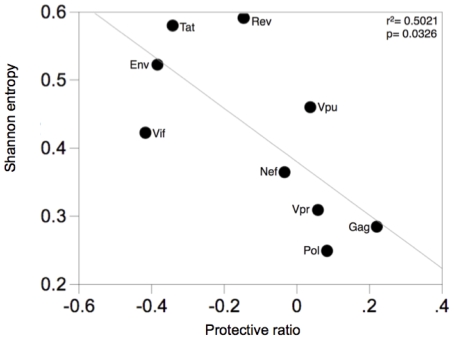
Relationship between CTL targeting and viremia. Protein-specific protective ratios were plotted as a function of the mean entropy of each HIV-1 protein. Protective ratios were calculated as the Log_10_ of the viral load of all the individuals who did not mount a CTL response against a protein over the viral load of all the individuals who had one or more CTL response(s) directed against that protein.

Our study focused on a previously described dataset [19], [24] that included viral genomic sequences, CTL response mapping, HLA-types and clinical data from 598 treatment naïve, chronically infected individuals from KwaZulu-Natal in South Africa; ∼99% of them were infected with HIV-1 subtype C. To initially assess the relationship between responses to viral proteins and viral loads, we calculated a “protective ratio” for each protein. This corresponded to the Log_10_ of the viral load of the individuals who had no CTL response against a protein, and the average viral load of the individuals who had at least one CTL response against that protein (Log_10_(VL non-responders/VL responders)). A positive protective ratio indicated that individuals recognizing the protein had lower viral loads than individuals who did not recognize the protein. Positive protective ratios were detected when having a CTL response against Gag and to a lesser extent Pol, Vpr and Vpu (Figure 1, x axis). In contrast, individuals mounting at least one response against other proteins, especially Vif, Env and Tat but also Rev and Nef, had higher viral loads than those who did not recognize those proteins (negative protective ratios). By comparing the protective ratios to the mean entropy values of each protein, we found a negative relationship, indicating that the most protective protein-specific CTL responses (with lower levels of viremia) corresponded to proteins of lower entropy: Spearman's correlation coefficient Rho ρ = −0.7167, p = 0.0298 (r^2^ = 0.5021, p = 0.0326) (Figure 1). In light of the beneficial role of Gag-specific CTL responses, we then characterized patterns of genetic variation in Gag sequences, particularly those linked to HLA-mediated mutations.

### Identification of co-varying residues in HIV-1 subtype C Gag

**Figure 2:**
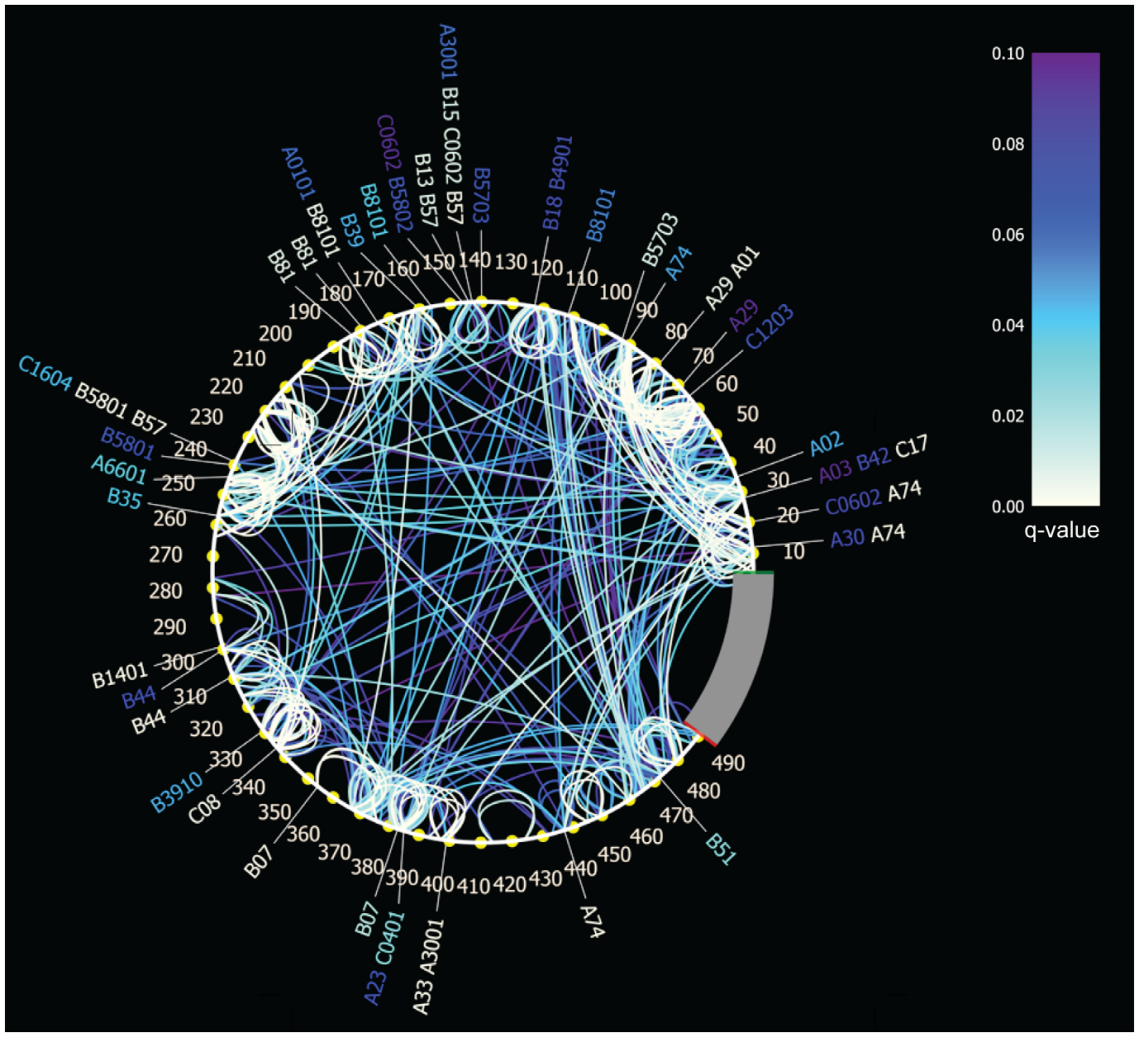
Amino Acid associations in HIV-1 subtype C Gag. Associations are depicted with a circular map: AA interactions among residues are represented with arcs, which are color-coded with a white to purple gradient – white corresponding to the strongest associations (i.e., lower q-values). HLA-restricted sites are identified by the HLA allele designations around the circle.

We determined AA interactions in Gag HIV-1 subtype C based on sequences and HLA type data obtained from 449 chronically-infected individuals. To correct for founder effects and HLA linkage disequilibrium, we identified associations using the program PhyloD [4], which we ran on 5 trees selected from a set of trees for their high log likelihood scores. Selecting the associations that met our congruence criteria (stable in the 5 tree topologies and q<0.1) resulted in a set of 727 associations: 69 (of the initial 74) HLA-restricted AA associations and 658 (of the initial 831) AA-to-AA associations. When symmetrical/reciprocal associations involving the same sites in the alignment were counted only once, we obtained 534 unique site-specific associations, which are displayed via connected arcs in a circle map (Figure 2). Compared to the distribution of AA in Gag, some AA were over- or under-represented among co-varying associations. Both lysine and threonine residues were found twice as often in AA-associations than expected from the AA distribution in Gag (two-tailed Fisher's exact test p = 0.006 and 0.030, respectively). In contrast, glycine and tryptophan were under-represented among co-varying residues (two-tailed Fisher's exact test p = 0.003 and 0.030, respectively). It could be logical that glycine and tryptophane were rarely detected among co-varying pairs, since they are less likely to be replaced by another AA given that they are the smallest and largest AA, respectively. Thirty-five of the co-varying sites were HLA-associated, including 30 sites located in p17/p24, 19 of which had been reported in a previous study of HLA-imprinted polymorphisms in p17/p24 [22]. HLA-mediated associations were evenly distributed in p17 and p24: 17 HLA-associations were identified in p17 and 26 in p24 (two-tailed Fisher's exact test p = 0.6134).

In this South African cohort, several alleles have been associated with lower viral loads in infected individuals (B13, B*3910, B*4201, B57, B*5801, B*8101), we therefore evaluated how these protective alleles were represented in our analysis. We found that a majority of HLA-associations were HLA class I B allele associations (54%) and half of these corresponded to known protective alleles. Although there was no difference in the distribution of associations between p17 (n = 3) and p24 (n = 10) (two-tailed Fisher's exact test p = 0.55) if we took into account the respective lengths of p17 and p24, the larger number of HLA-mediated associations in p24 may be associated with the efficient control of viral replication ascribed to p24-specific CTL responses.

### Distances among co-varying AA residues

**Figure 3:**
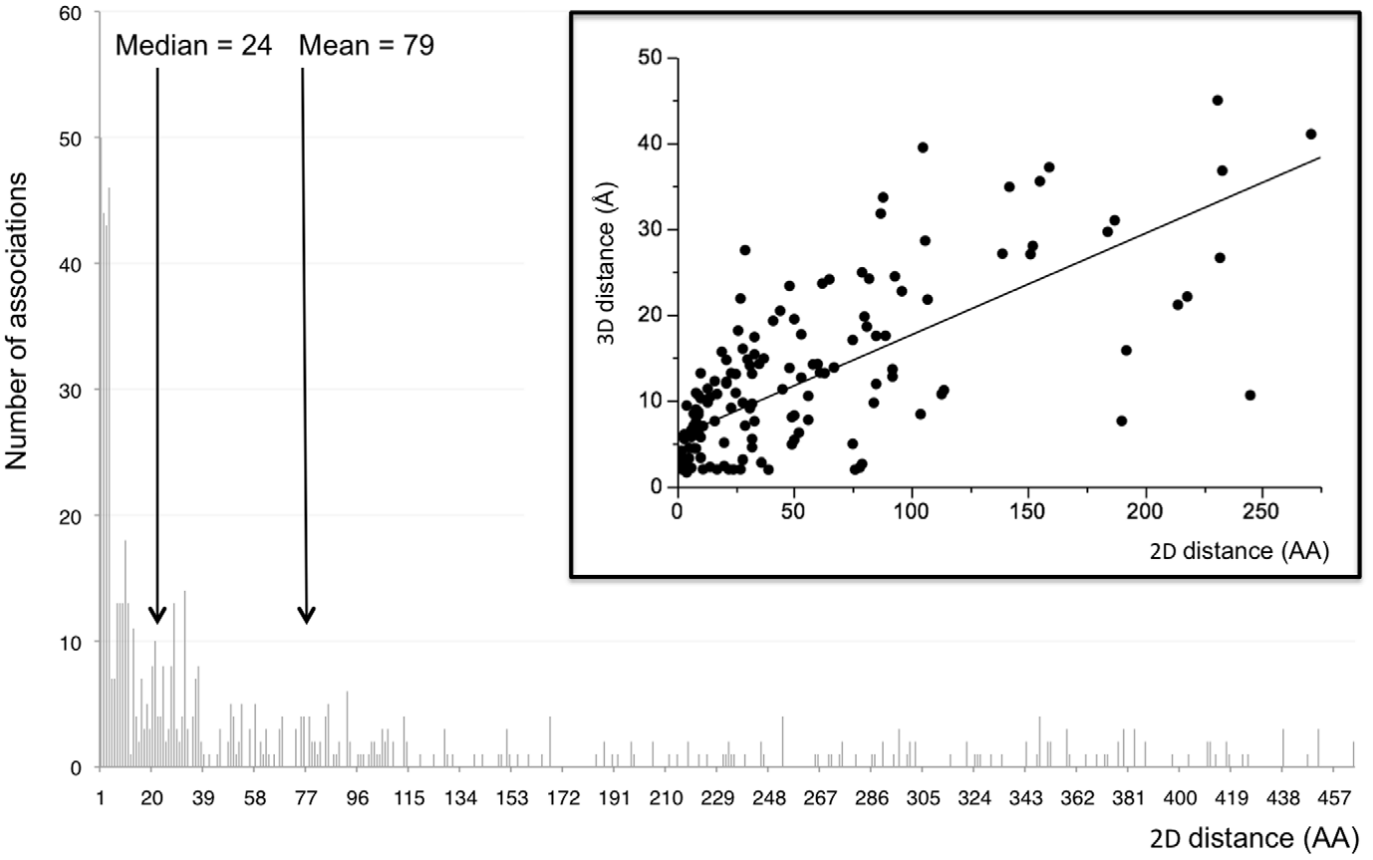
Distances between co-varying residues. The larger graph shows the distribution of the 2D distances between all the pairs of co-varying AA in Gag. The inset represents the relationship between the 2D and 3D distances.

A noticeable feature in the network of AA dependencies was the abundance of ‘neighboring’ interactions (Figure 2). Interactions between adjacent AA represented 40% of all unique associations, and those between residues that were no more than 3 AA apart represented 60% of all associations. Neighboring associations were significantly more frequent than expected by chance: p-value = 2.23e−27 for adjacent residues and p = 3.57e−58 for residues no more than 3 residues apart (based on random sampling of 10,000 associations and two-tailed Fisher's exact test) (Figure 3). Likewise, tri-dimensional distances (relative to a trimeric crystal structure of p17, and NMR structures for p24 and the p17/p24 polyprotein) showed a preponderance of short-range associations: residues separated by less than 5 Angströms (Å) corresponded to 27% of the associations in the trimeric crystal structure of p17 and 38% and 30% of the associations on the NMR structures of p24 and p17/p24, respectively. As the number of associations decreased with increasing physical distance, the significance (q-value) of the associations also decreased (Pearson's non-parametric ranked Rho ρ = 0.332, p<0.0001). Although interactions could be found up to 464 AA apart in the alignment, the median/mean linear distances between residues were 79 and 24 AA, respectively. In 3D structures, the median distance (of the minimum across all 3 structures) was 9 Å and mean distances were 10.2 Å in p24, 10.9 Å in p17 and 11.6 Å in the p17/p24 complex structure. Not surprisingly, there was a strong correlation between the 2D and 3D distances (Pearson's Rho ρ = 0.727, p<0.0001) (Figure 3).

Interestingly, most associations linked residues within a protein: 78% (195/251) of p17-associations involved residues both found in p17, likewise 77% (219/284) of p24-associations were amongst p24 residues. The number of intra-protein associations was significantly greater than the number of inter-protein associations, with p = 1.01e−10 for p17 and p = 2.33e−11 for p24 (Fisher's exact test). This shows that a majority of interactions were circumscribed within either p24 or p17, and conversely that a small number of associations might reflect physical interactions between the 2 proteins. To support that the associations between p17–p24 were not false positives, we found that: 1) there was no correlation between 2D and 3D distances for inter-protein associations (ρ = 0.2), while there was a positive relationship for intra- protein associations (ρ = 0.7 for both p17 and p24); 2) 3D distances for inter-p17-p24 associations were not larger than would be expected based on intra-p17 or intra-p24 associations (when correcting for larger 2D distances).

### Hubness in Gag subtype C

Another salient feature of the circle network of Gag dependencies is that only a fraction of residues were co-varying: all AA interactions were confined to 183 sites, whereas 309 ‘orphan’ sites were never involved in AA associations. An average of 5.4 residues were found at co-varying sites as opposed to 2.2 AA at orphan sites (p<0.0001), and the mean Shannon Entropy for co-varying sites was 0.594 as opposed to 0.168 for orphan sites (p<0.0001). Interestingly, sites that were both co-varying and HLA-restricted presented higher average Shannon Entropy (0.725) than other co-varying sites (0.565) (p = 0.036). Signals of co-variation and of adaptive evolution, as measured by dN/dS deviations, were detected simultaneously at certain sites: 39% of the co-varying sites (n = 49) had dN/dS >1, i.e., were under positive selection, while only 1 of the 260 orphan sites was under positive selection (p<0.0001, Two-Tail Fisher's exact test). More importantly, among co-varying sites under selection based on dN/dS, those that were also HLA-associated were more likely to be under positive selection (15/23) than those that were not HLA-associated (34/106) (p = 0.004, Two-Tail Fisher's exact test).

**Figure 4:**
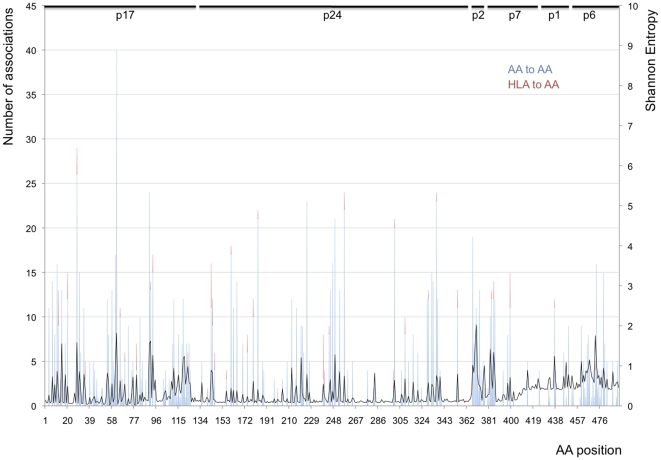
Hubness across HIV-1 Subtype C Gag. The number of co-varying partners and the Shannon Entropy are represented for each site along the Gag protein. The blue (lower) part of the bars represent the number of AA-to-AA associations and the red (upper) part of the bars represent the number of HLA-to-AA associations at each site. The secondary axis refers to the Shannon Entropy at each site in Gag (continuous black line).

A third noticeable characteristic of the circle map of AA dependencies is the higher density of associations in specific segments of Gag: a few sites, called hubs, have large numbers of connections, while most sites have 1–2 connections (Figure 4). For the co-varying sites (n = 183), the mean number of associations or hubness (H) was 7.1 (median = 5) (values correspond to all AA-to-AA associations only (not HLA-to-AA) to avoid the confounding effect of counting HLA-mediated pressure). Of the 35 HLA-associated sites, 30 of them had at least 5 interactions (maximum of 40). Hubness values were higher for co-varying HLA-associated sites (H_HLA_ = 9.9) than for co-varying but not HLA-associated sites (H_non-HLA_ = 6.4) (p = 0.0029). Thus, the fact that HLA-associated sites were more often co-varying, under positive selection (dN/dS >1), and had more AA connections suggests that HLA-mediated selection pressure may drive a substantial proportion of AA co-variation.

### Mutational pathways linked to variable sites

Variable sites can be differentiated by the representation of each AA at the site. An extreme example was the highly polymorphic site 28, which was under HLA A*03-mediated selection pressure (corresponding to the H28R mutation) and at which 10 AA states and 29 associations were found, revealing a sub-network of topologically close associations. Two A*03-restricted ‘best-defined’ epitopes span site 28 (RLRPGGKKH, RLRPGGKKHY), and mutations at position 28 (to residues R/Q/T) were shown to confer escape from A*03-restricted CTL responses [25].

Another scenario of Gag co-variation at variable sites corresponded to ‘toggle’ sites at which the two most common AA were found at relatively equal frequencies. One notable toggle case was position 357 in the HLA-B*07-restricted epitope GP(G/S)HKARVL, in which G (unadapted or ancestral AA) and S (adapted or escape form) were found in 51% and 49% of sequences, respectively, and individuals having either form presented comparable viral loads (Median: 47,800 (G) vs 35,850 (S) viral copies). The G357S mutation was associated with the A370T mutation (q-value = 2.93E−13).

Last, the most common pattern of AA representation at variable/co-varying sites corresponded to one AA being present in the majority of sequences, for example at position 146: A was present in 75% of the sequences examined, while P was found in 17% of sequences. The A146P mutation has been linked to the C-terminal B*57-restricted epitope ISPRTLNAW. Position 146, 147 and 149 were co-varying and the A146P mutation was linked to I147L. Interestingly, Draenert and colleagues [26] showed that A146P was an epitope processing mutation, and they noted that “it was unclear why the adjacent intra-epitopic mutation I147L was associated with HLA-B*57 expression while phylogenetic analysis had failed to demonstrate positive selection at this site”. Here, we showed that A146 was in repulsion with I147 (q = 0.005), and that B*57 was in attraction with P146 (q = 1.47E−07) and L147 (q = 0.007). This demonstrated that the AA pairs at positions 146 and 147 were co-varying and that there was indeed a B*57-association with position 147. As further evidence of the co-dependence of 147L and A146P, Troyer and colleagues [18] recently showed that A146P can considerably reduce viral replicative fitness, a cost that can be partially restored by I147L.

### Mutational pathways linked to sites under constraints

To utilize co-variation analyses to identify sequence constraints that could be exploited in a vaccine, we focused on the 66 associations linking conserved sites (i.e., sites where the consensus AA is present in over 85% of sequences), and analyzed the viral loads of the individuals with HIV-1 sequences that contained 1) the consensus residues at both co-varying sites, 2) a rare residue at both co-varying sites, or 3) only one site presented a consensus residue. Only a few sites showed significantly lower viral loads for individuals who presented rare residues at interacting conserved sites (Table 1), nonetheless, the trend toward lower viral loads may suggest that the presence of rare residues resulted in viruses with poorer replication capacities. Importantly, those 66 associations (between conserved sites) involved a total of 73 AA sites, of which 14 were HLA-associated; in comparison, there were 35 HLA-associated sites for the whole alignment of 492 sites, showing that associations between conserved sites were more likely to correspond to HLA-imprinted sites (Fisher's exact test 2-tail p-value = 0.0051), suggesting that some CTL-mediated escape mutations are deleterious. Therefore, associations between conserved sites may point to epitopes that are beneficial to target, due to their dependent fitness cost.

**Table 1:** Relationship between viral loads and co-varying associations linking conserved sites.

	n^a^			Median VL			% Decrease		Wilcoxon p-values	
Associations	CC^b^	RR^c^	CR^d^	CC	RR	CR	CC v. RR	CC v. CR	CC v. RR	CC v. CR
**12K-20R**	314		61	37,500		55,100		47		0.749
**20R**-68L	323	8	44	37,800	52,100	67,650	38	79	0.778	0.565
**31L**-118A	289		77	38,300		34,800		−9		0.992
**35V**-39R	336	8	31	41,935	62,150	19,300	48	−54	0.745	**0.01**
42E-75L	341	2	32	39,600	1,593	32,350	−96	−18	**0.033**	0.231
50L-82V	351	2	23	42,300	20,800	7,050	−51	−83	0.468	**0.006**
60I-82V	347	5	25	45,200	4,830	17,300	−89	−62	**0.016**	**0.038**
82V-84T	329	10	37	40,000	5,210	36,100	−87	−10	**0.011**	0.978
**149P-159I**	343	3	28	37,900	271,000	44,350	615	17	0.67	0.787
**159I**-385G	332	1	41	37,500	157,000	47,800	319	27	0.368	0.87
55E-**163A**	302	11	61	37,850	18,700	42,300	−51	12	0.871	0.667
**163A**-165S	336	25	13	38,250	56,400	13,500	47	−65	0.65	0.201
**163A**-191V	337	5	32	37,900	56,900	39,750	50	5	0.978	0.873
**163A**-168V	311		63	38,300		37,200		−3		0.982
**163A-182Q**	307	1	66	37,900	67,500	46,800	78	23	0.77	0.978
**163A-242T**	303	14	57	42,100	42,300	26,000	0	−38	0.731	0.052
**163A**-267I	338	2	34	37,850	69,300	46,800	83	24	0.678	0.672
**20R**-165S	314	9	51	37,900	13,500	47,800	−64	26	0.676	0.944
**177E-186T**	347	8	19	40,900	51,300	13,700	25	−67	0.548	**0.029**
**182Q-186T**	319	5	50	38,300	9,470	49,550	−75	29	**0.041**	0.535
**182Q**-267I	337	3	34	37,900	51,300	65,850	35	74	0.768	0.911
116Q-**186T**	337	3	31	40,000	107,000	24,200	168	−40	0.248	0.185
165S-**186T**	324	5	45	41,335	13,700	25,700	−67	−38	0.504	0.102
173T-**186T**	337	8	29	40,000	85,650	11,300	114	−72	0.704	**0.031**
**186T**-190T	346	10	18	40,885	42,450	16,700	4	−59	0.407	**0.029**
**186T**-384K	335	3	33	42,100	37,800	22,700	−10	−46	0.873	0.136
218V-219H	313	8	53	37,900	179,500	35,080	374	−7	**0.028**	0.505
219H-**332T**	286		88	38,100		38,000		0		0.207
219H-**247I**	305	6	63	37,900	71,540	47,800	89	26	0.238	0.166
**247I**-250M	322	8	44	37,450	255,000	37,550	581	0	**0.009**	0.885
**247I**-255P	341	4	29	37,900	128,000	47,800	238	26	0.3	0.084
224A-225P	351	10	13	37,100	140,500	111,000	279	199	**0.038**	0.61
285I-**303T**	316	3	52	38,950	45,000	27,400	16	−30	0.892	0.505
**303T**-323V	291		80	37,900		47,050		24		0.995
**303T**-377M	281	12	78	40,000	52,150	23,250	30	−42	0.839	0.064
**303T**-310T	313	5	53	39,600	13,200	45,000	−67	14	0.33	0.596
331K-**332T**	324	2	45	36,350	8,720	59,200	−76	63	0.203	0.133
118A-**332T**	300	7	61	39,150	47,800	33,800	22	−14	0.432	0.621
127K-**332T**	302	5	64	37,900	47,800	37,500	26	−1	0.966	0.519
**332T**-336A	311	23	40	38,200	99,000	30,050	159	−21	0.122	0.399

Shown are associations that involved an HLA-associated site (in bold) or at which a mutation had a significant impact on viral loads.

a Number of individuals.

b Consensus AA at both co-varying sites.

c Rare residues at both co-varying sites.

d Consensus AA at one site and a rare AA at the other co-varying site.

For example, in the B*57-restricted epitope KAFSPEVIPMF, the A163G CTL escape mutation incurs a fitness cost *in vitro* (A is found in 89% of sequences), a cost that could partially be restored by the S165N compensatory mutation [16]. The 4th strongest association (q-value = 2.03e−20) in our analysis showed that the presence of G163 (found in 8% of sequences) was associated with the S165N mutation (found in 9% of sequences), demonstrating that our method identifies associations between deleterious and compensatory mutations. In addition, individuals who had one rare residue had lower viral loads than individuals with consensus residues at both sites, while individuals with both rare residues, i.e., with the escape and the compensatory residue, had higher viral loads than individuals with consensus residues at both sites (Table 1).

**Figure 5:**
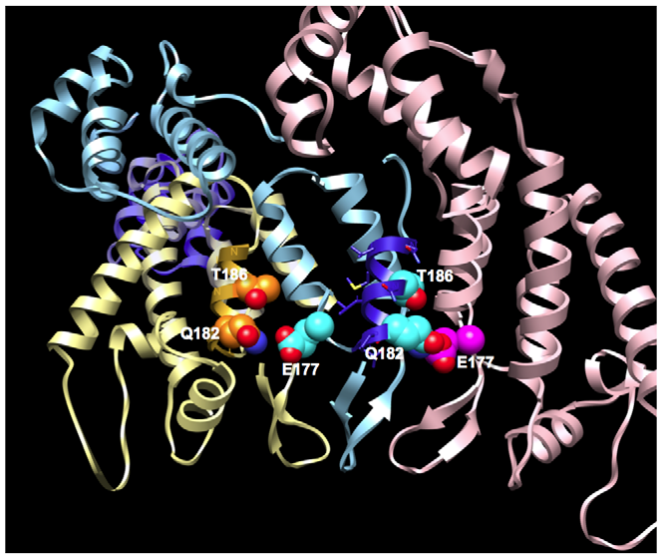
Sub-network associated with the HLA-B*81 epitope TPQDLNTML. The tridimensional structure shows three p24 chains of HIV-1 subtype C (colored in light blue, golden yellow and pink) and the epitope TPQDLNTML is highlighted in bright blue on chain A. Side-chain atoms for the residues E177, Q182 and T186 are represented in sphere format.

**Figure 6:**
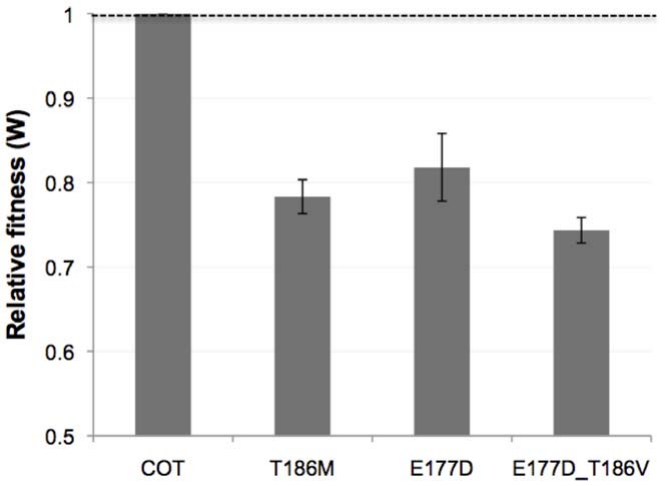
Fitness competition assays between viruses mutated at residues in the sub-network associated with the HLA-B*81 epitope TPQDLNTML. The relative fitness of viruses presenting a mutation at site 177, 186 or at both sites is compared to that of the wt COT virus. Fitness competition assays were performed against a wt COT virus; the proportion of viral RNA from the mutant and wt viruses was measured at day zero, three and five (see methods).

HLA-B*81 was associated with polymorphisms at positions 177 (E found in 97% of sequences), 182 (Q in 88%) and 186 (T in 91%); both residues 182 and 186 are embedded in the epitope TPQDLNTML (B*8101) (Figure 5). There was a positive relationship between the consensus residues T186 and E177, while there was a negative relationship between T186 and the adapted E177D (q-value = 2.8e−06). Figure 5 shows three p24 chains of HIV-1 subtype C and highlights the epitope TPQDLNTML and particularly the residues E177, Q182 and T186: while Q182 and T186 align with each other and are located on the same face of the helix, E177 appears to interact with the residue Q182 of the neighboring chain. Interestingly, individuals presenting viruses with rare residues at one position (177 or 186) had significantly lower viral loads than those who possessed the consensus T186 and E177 (Median VL Consensus+Consensus = 40,900, Median VL Consensus+Rare = 13,700; p = 0.029). The decreased viral loads in the presence of a rare residue hints at a fitness cost, an hypothesis supported by the reduction in infectivity (of ∼29-fold) reported by von Schwedler and colleagues with the E177A mutation [27]. Moreover, we performed *in vitro* fitness competition assays between viruses with the consensus or the mutant residue and found that: mutations to a rare residue at sites 177 and 186 both had a significant fitness cost, a cost that was amplified when residues were mutated simultaneously (Figure 6). In addition, we found negative associations between the adapted AA at positions 182 and 186; in agreement, individuals presenting viruses with rare residues at both positions 182 and 186 had significantly lower viral loads than those who possessed consensus residues (Median VL Consensus+Consensus = 38,300, Median VL Rare+Rare = 9,470; p = 0.041), suggesting that both escape mutations may not occur simultaneously except in debilitated viruses.

## Discussion

Our results showed that CTL responses against Gag (as opposed to responses against other proteins) were fundamental to the control of viral replication in a cohort of HIV-1 subtype C infected individuals in KwaZulu-Natal, South Africa, and also suggested that control of viremia was associated with the reduced variability of the Gag protein. Analysis of co-variation in HIV-1 Gag subtype C showed intricate patterns of dependent mutations associated with CTL-driven polymorphisms.

Our co-variation results were derived with a method that can overcome traditional challenges for co-variation studies. In particular, it sets apart co-variation due to selection pressure from co-variation due to shared ancestry by modeling multiple interactions simultaneously. Moreover, it is conditioned on the HLA types observed in each subject, allowing us to identify pathways of particular interest to CTL escape. Nonetheless, there are limitations to the identification of co-variation. For example, associations between two AA that are in different epitopes restricted by the same HLA allele may be a consequence of immunodominance patterns, i.e., the preferential or time-ordered targeting of epitopes. A second issue is the noise in the data; yet, we note that the fuzziness of the network is inherent to the fast pace of HIV evolution and the cross-sectional nature of our analysis. It may also point at biological differences between permanent and transient interactions.

The circle map of dependencies illustrated the topological clustering of AA interactions in Gag, underlining the preponderance of ‘neighboring’ interactions. The distance between co-varying residues suggests two types of interactions: i) structural interactions with combined replacements between spatially proximal AA to maintain the local structure stability, and ii) functional interactions between pairs of distal AA to connect distant functional determinants.

It has been shown in protein-protein interaction studies that essential genes have on average more partners, i.e., higher hubness [28]. By analogy, one might expect that hubs in Gag might have a greater impact on viral fitness, since a disturbance at a highly connected residue would probably be more detrimental to the virus than a change at a lowly connected site. Accordingly, we found that HLA-associated sites had conspicuously high numbers of associations and some CTL escape mutations at those HLA-restricted sites are known to have a fitness cost [16], [18], [21], [29], [30], [31]. By co-analyzing HLA-imprinting and AA-to-AA interactions in Gag, we described examples of evolutionary constraints that are forced upon some residues and elucidated some mutational pathways associated with HLA-restricted polymorphisms, for example, linked to the B*57-restricted epitopes KF11 and IW10.

A key outcome of our analysis is our ability to stratify the dispensability of particular sites, based on their dependent mutational pathways, and thereby to better understand the relationship between AA mutability and viral fitness. Hence, our CTL-driven mutational dynamics maps reflect the lethality profile of the protein, and we have found some relationships between the presence of rare residues at co-varying sites and lower viral loads for the infected individuals. This pertains to recent vaccine strategies that have proposed to focus on conserved elements of HIV-1 [1], [32]. The sites that remain conserved despite the influence of selective forces are of specific interest because they correspond to the ‘Achille's heel’ of the virus and are attractive anti-viral or vaccine targets. We previously proposed a CTL-based ‘Conserved Element’ (CE)-vaccine that would be composed of conserved elements of the HIV-1 proteome [1], [32], based on the rationale that ‘an efficacious vaccine must elicit responses toward HIV-1 segments that cannot mutate without severely compromising viral viability, and must not elicit responses against variable, immunodominant decoys’. Here, we present a strategy to expand ‘conserved elements’ while still blocking CTL escape paths by taking into account CTL-driven mutational pathways. Combining results from replicative fitness competition assays and from analyses of the effect of mutations on viremia to co-variation data provides ways to extend CE beyond their minimal length (which is a consequence of their rigid degree of conservation). For example, the vaccine insert could include one or both residues of a co-varying interaction in order i) to link residues that are known to incur a high fitness cost when mutated or ii) to avoid including pairs of residues that can compensate the fitness cost associated with a mutation. In addition, certain HLA-associated hubs under constraints with multiple connections underline sites that may represent vulnerable spots for HIV and that can be incorporated in a vaccine construct.

## Materials and Methods

### Cohort and data

Study participants were antiretroviral drug naïve adults from Durban, KwaZulu-Natal, South Africa. Several studies on this cross-sectional cohort have been published [16], [19], [22]; Host (Viral Loads, CD4 counts, HLA types, ELISpot mapping of CTL responses) and viral genetic details are also available at: http://www.hiv.lanl.gov/content/immunology/hlatem/index.html.

Near full-length genomes were PCR-amplified, cloned and sequenced from plasma-derived RNA samples for 272 individuals [22], [33]. Targeted sequencing of the Gag gene for other study participants was performed likewise (n = 36) [22] or by population sequencing as previously described (n = 141) [16], [34], [35].

### Phylogenetic Analysis

We included one HIV-1 sequence per individual and excluded sequences that were non-subtype C, inter-subtype recombinants or hypermutated, based on inspection of alignments and phylogenetic trees and analysis of pairwise distances among sequences in the dataset. Recombination was evaluated based on Rousseau et al. [24], a study that included a subset of the present dataset. The resulting dataset included 449 HIV-1 Gag subtype C sequences, to which 12 non-subtype C reference sequences from the Los Alamos HIV Sequence Database (LANL) were added as an outgroup to root the trees. Nucleotide sequences were aligned with ClustalW version 1.8 [36] and manually edited with MacClade v4.08 [37], resulting in a 1476-nucleotide (nt)-long alignment after stripping ‘gap’ columns that resulted from insertions found in a single individual. PAUP* [38] was used to generate 500 parsimony trees. These were used as input in PhyML [39] to reconstruct a set of maximum likelihood trees by estimating and implementing the GTR + I + G nucleotide substitution model. We selected 5 trees with the highest log likelihood scores for subsequent determination of AA associations.

### Identification of AA-to-AA and HLA class I to AA associations

Co-variation analyses were performed using a recent implementation of the method of Carlson et al. [4]. This approach fits a Phylogenetic Dependency Network (PDN) to the data. Each node in the network represents a binary variable corresponding either to a specific residue at a given AA site or the presence or absence of an HLA allele. For each target AA node, the algorithm identifies the set of other AA and HLA nodes that significantly predicts the state of the target node, with the resulting probability distribution conditioned on the phylogeny. For each pair of nodes that are associated, there are four possible statistical relationships: attraction is defined as the association of the presence of a particular predictor variable (AA or HLA allele) with the presence of a target variable (AA or HLA allele). Conversely, repulsion is defined as the association of the presence of a predictor variable and the absence of a target variable. Escape is defined as the association of the absence of a predictor variable and the absence of a target variable, while reversion is defined as the absence of a predictor variable and the presence of a target variable. The significance of each association is quantified using a likelihood ratio test, calibrated for multiple testing using q-values [40]. Co-variation analyses performed independently on the 5 ‘best’ trees yielded a total of 905 significant associations (q<0.1, corresponding to a false discovery rate of 10%), ranging from 794 (tree 4) to 827 (tree 3) associations per tree. We selected for analysis only the 727 associations that were concordant across all of the tree topologies.

### Characterization of the set of associations

Distances between interacting residues were calculated based on available tri-dimensional Gag structures, with 3 types of measurements: within p17, within p24, and in a p17/p24 complex. Since p17 is a trimeric structure, distance measures between residues may correspond to residues that are on different molecules in the trimer. Additionally, p17 data was derived from a crystal structure, thus distances tend to be higher than for the p24 and p17p24 structures, that were obtained by NMR and thereby include the hydrogen atoms. Reported are the minimum distances found over the 20+ models.

Shannon entropy scores were calculated with the Entropy1 program (www.hiv-web.lanl.gov/content/hiv-db/ENTROPY/entropy.html).

### Detection of Selection

Rates of nonsynonymous (dN) and synonymous (dS) mutational changes across codon sites were estimated using the Single Likelihood Ancestor Count (SLAC) [41] approach under the GTR nucleotide substitution model with a significance level threshold of 0.05, as implemented in Hyphy [41] (www.hyphy.org).

### Fitness competition assays

#### Viruses

The HIV-1 Subtype B Center of Tree (COT) Gag-p24 sequence was placed in an NL4-3 backbone using the restriction sites *BstEII* and *SfiI*, which were generated in a Gag-p24 COT expression plasmid and a pNL4-3 infectious clone. Point mutations were engineered using the QuikChange XL site-directed mutagenesis kit (Stratagene). The complete HIV-1 coding region of the variant viruses were PCR-amplified and sequenced.

#### Cells, titration of chimeric viruses

CEMx174 cells were cultured in appropriate medium supplemented with 10% fetal bovine serum. Viral stocks were generated by transfection of HEK293T cells with 1 µg of plasmid DNA using Fugene (Roche). Supernatants were harvested 48 h after transfection, and frozen aliquots were stored at −80°C. Titers were determined in CEMx174 cells using the method of Reed and Muench. The capsid concentration of the viral stocks was quantified by p24 enzyme-linked immunosorbent assay (ELISA).

#### Viral replication assays

CEMx174 cells were infected with wt or variant viruses or both; mono- and dual-infections were done in triplicate. Viruses were added at an MOI = 0.005 to 10^5^ cells, and washed 24 hr post-infection. Viral production was monitored with p24 ELISA and aliquots of supernatants were sampled for 6 days. Viral RNA was extracted from supernatant aliquots, cDNA synthesis was done using SuperScriptIII (Invitrogen) and genes of interest were amplified by PCR and fully sequenced to quantitate the proportion of the variant and wt viruses by measuring peak height (using an in-house measurement program). Following propagation, we sequenced the entire viruses to verify that no reversion or additional mutations occurred outside of the gene of interest. The fitness of the variant viruses relative to the wt viruses were calculated using the method described by Wu and colleagues [42].
